# Children's Judgments of Possibility Align With Their Judgments of Actuality

**DOI:** 10.1111/desc.70084

**Published:** 2025-10-22

**Authors:** Mopreet Pabla, Andrew Shtulman, Ori Friedman

**Affiliations:** ^1^ Department of Psychology University of Waterloo Waterloo Canada; ^2^ Department of Psychology Occidental College Los Angeles California USA

**Keywords:** actuality, immoral actions, improbable events, modality, possibility

## Abstract

Children often say that possible events are impossible, and only gradually come to see these events as possible. For instance, they often deny that people could do unusual things, like own a pet peacock, or immoral things, like stealing or lying. These possibility denials are surprising. For instance, children have first‐hand experience with the very moral violations they say are impossible. In two experiments (total *N* = 220), we provide evidence that children's possibility denials can nonetheless be taken at face value and do not merely arise from quirks in how children understand questions about possibility. We do this by showing that these denials also arise in children's judgments of actuality—their judgments about what has actually happened and about which assertions of actual events could be true. In Experiment 1, children aged 4–7 judged whether ordinary, immoral, and improbable events could happen or had ever happened. With both judgments, children mostly responded affirmatively to ordinary events, often responded negatively for immoral events, and mostly responded negatively to improbable ones. In Experiment 2, children aged 5–7 judged if assertions of the three kinds of events could be true, and the same pattern emerged again. Together, these findings show that children's denials of immoral and conceptually improbable events extend beyond their judgments about what is possible and match their inferences about what is actual. These correspondences across judgments suggest that children drew on a single procedure, or set of procedures, for assessing possibility.

**Summary:**

- We show that children judge unexpected events as both impossible and nonactual.
- Four‐ to seven‐year‐olds judged if events could happen, if events had ever happened, and if assertions about events could be true.
- For all judgments, children often responded negatively to immoral events, and mostly responded negatively to conceptually improbable ones.
- Children's possibility denials can be taken at face value, and do not reflect quirks in how they respond to questions about possibility.

## 1 Introduction

Judgments about which events are possible change with age and in a surprising way. Children are widely believed to be more imaginative and prone to fantastical thinking than adults, and therefore likely to see a wider range of events as possible (see Shtulman 2023 and Woolley and Ghossainy 2013 for discussion). But this belief is wrong; young children deem many possible events impossible and only come to see these events as possible with development. In contrast with adults, children aged 4–8 often deny the possibility of conceptually improbable events like drinking onion juice or owning a pet peacock (e.g., Shtulman and Carey 2007). They also deny that people can violate social norms and moral rules, for instance, by stealing or lying (e.g., Chernyak et al. 2013; Shtulman and Phillips 2018).

At face value, children's denials reflect their beliefs about what is possible. When children deny that a person could own a pet peacock or that a person could steal someone else's property, it is because they really believe that these events could not happen. In this paper, though, we adopt the perspective of skeptics, and question whether children's denials can be taken at face value. For this reason, we develop a skeptical alternative to the face value account, which holds that children's denials do not reflect their true assessments of possibility and instead arise from quirks in how they understand test questions. We then test between the accounts by assessing children using two new measures which focus on children's judgments of *actuality*—their judgments of what has actually happened and which assertions about actual events could be true. Testing between the accounts, then, might reveal that children's denials are not what they appear to be. Alternatively, it could broaden evidence for the face value account.

### 1.1 Young Children's Possibility Denials

Children's denial of the possibility of improbable events was first revealed in an experiment where 4–8‐year‐olds were asked whether a person in real life could perform various actions (Shtulman and Carey 2007). Children denied that the person could do impossible things like walking through a wall and eating lightning for dinner. But children also denied that the person could do improbable things like having a lion as a pet and making a building shaped like a coffee mug. At Age 4, children denied that improbable acts could happen nearly as often as they denied this for impossible ones; at Age 6, they denied that improbable events could happen about half the time; and even at Age 8, they did not affirm possibility as often as adults did.

These results have been replicated and extended, both in Western cultures (e.g., Goulding et al. 2024; Goulding and Friedman 2020; Lane et al. 2016; Nancekivell and Friedman 2017; Nolan‐Reyes et al. 2016; Shtulman 2009; Shtulman and Phillips 2018; Shtulman et al. 2023; Weisberg and Sobel 2012) and elsewhere (Nissel et al. 2024; also see Davoodi et al. 2023 and Payir et al. 2021). Children's skepticism is also relatively robust to attempts to help them recognize that improbable events can happen. They are slightly more likely to admit that these events are possible when provided with certain forms of testimony affirming the events did happen (Lane et al. 2018; also see Danovitch and Lane 2020) and when asked about events taking place in distant countries (Bowman‐Smith et al. 2019); but in neither case do children predominantly affirm possibility. From Ages 4 and 5, children are also more likely to affirm that improbable events can happen if first told about similar events or, under some circumstances, if first informed about causal mechanisms that could bring the events about (Goulding and Friedman 2021; Goulding et al. 2022; Shtulman et al. 2024). But even this information does not bring children to affirm possibility at ceiling.^1^

Young children also sometimes deny that people can violate social norms and moral rules (e.g., Browne and Woolley 2004; Kalish 1998; Shtulman et al. 2023). In one study on 3–10‐year‐olds, younger children denied that people could behave unconventionally (e.g., wearing pajamas to school) and immorally (e.g., lying to a parent) for about half the transgressions they were asked about (Shtulman and Phillips 2018). Similarly, in another study, 4–11‐year‐olds in the United States and Nepal (Chernyak et al. 2013) often judged that agents who normally heed social rules (e.g., saying nice things to friends) could not choose to violate the rules (e.g., saying something mean to a friend). These denials were common for younger Americans and for Nepalese children across the full age range; only older American children judged that the agents could violate social rules. Even adults sometimes deny that people can violate norms: Although adults normally affirm that immoral actions are possible, they sometimes deny the possibility of such actions when forced to respond quickly (Acierno et al. 2022; Phillips and Cushman 2017).

### 1.2 Two Accounts

Taken at face value, children's possibility denials suggest that they judge improbable and immoral events to be impossible. On this view, judgments of what is possible are initially quite constrained and only broaden with age; in thinking about what can happen, children see fewer events as possible than do adults. Several alternative proposals have been offered about how children make these judgments—for instance, they might try to envisage how events could come about, or ask themselves if they know of similar events having happened (e.g., Goulding and Friedman 2021; Lane et al. 2016; Shtulman and Carey 2007; for an overview, see Shtulman 2023). Children's denials of social and moral violations further suggest that possibility representations are strongly influenced by notions of permissibility. Possibility and permissibility representations may not be fully differentiated (Phillips and Knobe 2018; Shtulman and Phillips 2018).

There are reasons, though, to doubt whether children's denials should be taken at face value. Consider first children's judgments about improbable events. Although children often deny that these events can happen, many findings suggest children see improbable events differently than they see truly impossible ones: Children aged 6–8 report difficulty visually imagining impossible events, but no difficulty imagining improbable ones (Lane et al. 2016). Children aged 5–9 take longer to assess the possibility of improbable events compared with both impossible ones and ordinary events too (Goulding et al. 2024). When 4‐year‐olds construct stories, they prefer including improbable events over impossible ones (Weisberg and Sobel 2012). Some manipulations that affect children's acceptance of improbable events do not correspondingly affect their acceptance of impossible ones (e.g., Bowman‐Smith et al. 2019, Experiment 2; Lane et al. 2018). These differences would not be expected if children saw improbable events as truly impossible.

Turning to social and moral violations, there is a much stronger reason to question whether children think these events are impossible: Whereas children are unfamiliar with the improbable events they have been asked about, they have first‐hand experience with the kinds of social and moral violations they say are impossible. Young children themselves lie, cheat, steal, and hit (e.g., Evans and Lee 2013; Nucci and Turiel 1978; Zhao et al. 2018). Children as young as Ages 2 and 3 often protest and intervene when they observe others’ social and moral transgressions (e.g., Rakoczy et al. 2008; Riedl et al. 2015; Rossano et al. 2011). They also assess the wrongness of violations and whether these warrant punishment (e.g., Smetana et al. 1993; for reviews see Marshall and McAuliffe 2022 and Schmidt and Rakoczy 2023), anticipating the emotions felt by transgressors, victims, and bystanders (e.g., Arsenio and Kramer 1992; Nunner‐Winkler and Sodian 1988). Given children's first‐hand experience with social and moral violations, it is difficult to see how they could view these actions as impossible.

These doubts about taking children's denials at face value might be explained by an alternative account, which holds the denials arise because of how children interpret questions about possibility. In almost all studies, children have been asked questions using the modal term *could* (e.g., “Could a person find an alligator under the bed in real life?”). Modal terms, though, can refer not only to what is physically or logically possible but also to what is permissible or ought to happen (Kment 2021; Shtulman and Tong 2013). For instance, saying, “I could not leave the room” can either indicate a true inability to leave (e.g., being locked inside) or that leaving was impermissible (e.g., because of work duties). For this reason, the questions are potentially ambiguous, as children might *sometimes* interpret the questions as asking about permissibility. For instance, when asked if a person could have a pet peacock, children might simultaneously consider whether this is possible and whether it is permissible (e.g., whether people ought to have pet peacocks). Adults may also sometimes interpret similar questions as asking about permissibility. As noted above, some recent experiments have found that when adults were put under time pressure and judged whether different actions are possible or impossible, they became more likely to deny the possibility of immoral actions. However, even when there was no time pressure, and adults were, on the contrary, asked to deliberate, they still indicated that immoral actions are impossible more than 25% of the time (Acierno et al. 2022). These denials for deliberative responses suggest that adults sometimes saw the question as asking about permissibility rather than physical or logical impossibility.

The ambiguous meaning account does not posit that children always misunderstand “could” questions or that they always see these questions as asking about permissibility. We know that children as young as Age 4 often interpret “could” questions as asking about what is physically possible, for instance, from their responses when asked whether a person who drew a red marble from a bag of red and blue marbles could have drawn a blue one instead (Shtulman and Carey 2007; Nissel et al. 2024). The account only posits that children might waver between interpretations in situations where the meaning is ambiguous—that is, in situations where norms have been violated. The account applies most directly then to children's denials of social and moral violations. But it might also speak to children's denials of improbable events, as these events almost always involve people doing irregular things (e.g., drinking pickle juice, having a pet lion or peacock) which children might think *ought* not happen for prudential reasons.

### 1.3 Adjudicating Between the Theories

The accounts offer competing explanations for children's denials of questions about what could happen in real life. They differ, though, in how they predict children will respond to other questions about possibility—questions with different phrasings that cannot be interpreted as asking about what ought to happen or what is permissible. The face‐value account predicts that children's denials will continue for improbable events and for social and moral violations. By contrast, the ambiguous meaning account predicts that children will now affirm that these events are possible.

Some previous work speaks to these opposing predictions. In this work, children were asked if events would require magic to happen. This question probes beliefs about possibility since magic is only needed for impossible events. But the question cannot be interpreted as asking about permissibility. At first glance, the findings more clearly fit the face‐value account, as children have often said that magic would be required for both improbable events (Nissel et al. 2024, Study 2; Shtulman and Carey 2007, Experiment 3; Shtulman and Phillips 2018, Study 2) and for social violations (Browne and Woolley 2004; Shtulman and Phillips 2018, Study 2).

Nonetheless, the findings are not entirely clear‐cut. One reason is that affirmations that events require magic are less frequent than possibility denials with *could* questions; most studies have found that children only affirm that magic is required for improbable events and social violations at chance rates or less often (e.g., Browne and Woolley 2004; Nissel et al. 2024; Shtulman and Phillips 2018). Also, in one recent study, 4–8‐year‐olds sometimes spontaneously offered magic as an explanation for impossible events but *never* did so for improbable events (Nissel et al. 2024). This same study also observed mixed findings when looking at correlations between magic judgments and *could* judgments. Together, these findings raise the worry that children do not truly believe that improbable events and social violations *require* magic. Children's responses might instead indicate that magic *can* produce unusual and unsavory outcomes (without being necessary for these outcomes). Moreover, the question “would it take magic?” introduces ambiguities of its own, as children may understand magic as referring to illusions, outcomes possible only in fiction, or outcomes that defy explanation but are still possible (Phelps and Woolley 1994; Rosengren et al. 1994).

### 1.4 The Current Experiments

We tested the accounts by using questions that cannot be interpreted as asking about what should happen or what ought to happen. To this end, we turned to questions about actuality. In Experiment 1, we first asked 4–7‐year‐olds whether events had ever happened, and contrasted these with responses to questions about whether events could happen. For example, we asked children whether someone had ever eaten pickle‐flavored ice cream in real life or whether someone could eat pickle‐flavored ice cream in real life. Judging whether an event has ever happened is different from judging whether the event is possible. Many possible events have never happened—some will happen in the future, and others may never happen at all. Even so, judgments of what has happened index possibility, since all events that have happened are possible.

In Experiment 2, we then asked 5–7‐year‐olds about whether utterances could be true. For instance, in one trial, children were told, “*Somebody said they had a pet peacock*,” and were then asked, “*Could that be true?*” This question is again informative about the possibility, but cannot be interpreted as asking about whether events ought to happen.^2^ This experiment resembles two previous experiments that probed children's possibility judgments about claims asserted by informants (Danovitch and Lane 2020; Lane et al. 2018). Children in those experiments, though, were not asked about whether the testimony could be true. Instead, they were asked about the possibility of the events themselves, as in almost all previous work.

In both of our experiments, we asked children about three kinds of events as follows: ordinary, improbable, and immoral. The face value account predicts that children should respond to our two novel questions much as they have responded to questions about whether various events could happen in real life. For instance, it predicts that children will say that ordinary events have happened in real life, but improbable and immoral events have not. After all, if children think that having a pet peacock is impossible, they should deny that anyone has ever had one.^3^ By contrast, the ambiguous meaning account predicts that children will say that many improbable and immoral events have happened, similar to ordinary events, and that assertions about all three events could be true.

The accounts could also each get mixed support. For instance, the face value account might be supported for improbable events, while the ambiguous meaning account is supported for immoral ones, given that children have seen or experienced many of the immoral events but have neither seen nor experienced any of the improbable events. Finally, it is important to acknowledge that the accounts are not exhaustive. Even if children do not misinterpret questions about what could happen as suggested by the ambiguous meaning account, there might be other reasons their responses should not be taken at face value.

## 2 General Methods

Data and code can be downloaded at the Open Science Foundation at https://osf.io/dx568/. We report all measures, manipulations, and exclusions.

In each experiment, we aimed to test 20 children per age‐in‐year in each between‐subjects group, though oversights led to slight departures from this goal. We chose this target sample size as it has sufficed to reveal significant effects in past work on children's judgments of possibility. For instance, samples of 20 children per age in years were also used by Nissel et al. (2024) and by Tipper et al. (2024). Almost all children were recruited and individually tested in their schools and childcare centers in Waterloo Region, Ontario, Canada, with just a few children in the first experiment (5 out of 159) tested online via Zoom. Demographic information was not collected from each child as per the allowances of our IRB. However, a recent census found that 64% of residents in the region are White, and South Asians are the largest visible minority; these figures were also corroborated by a census of students in the Waterloo Region District School Board, where much of the data was collected. Beyond this, the area is mostly suburban and predominantly middle‐class.

For children tested in person, the experimenter showed the testing materials on a laptop computer. For the few children tested online, the experimenter shared her screen to show the same materials. We analyzed all results using generalized estimating equations (GEEs) models. We used GEEs because they account for within‐subject correlations introduced by repeated measures (see Frank et al. 2025), and their results can be readily outputted to Type III tests, similar to what is commonly reported in analysis of variance (ANOVA). In each experiment, we ran a single omnibus model testing for all main effects and interactions, much as if we had run an ANOVA (i.e., we did not add or drop terms). We ran the models in R using *geepack* (Højsgaard et al. 2006) and used the joint_tests function in *emmeans* (Lenth et al. 2019) to yield Type 3 outputs and to run post hoc tests.

## 3 Experiment 1

### 3.1 Methods

**Participants**. We tested one hundred and fifty‐nine 4–7‐year‐olds (*M*_age_ = 6;0, range = 4;1–7;11, 77 female and 82 male). An additional eight children were seen but excluded from analysis: four because of experimental error, and four because they responded to fewer than half of the test questions.

**FIGURE 1:**
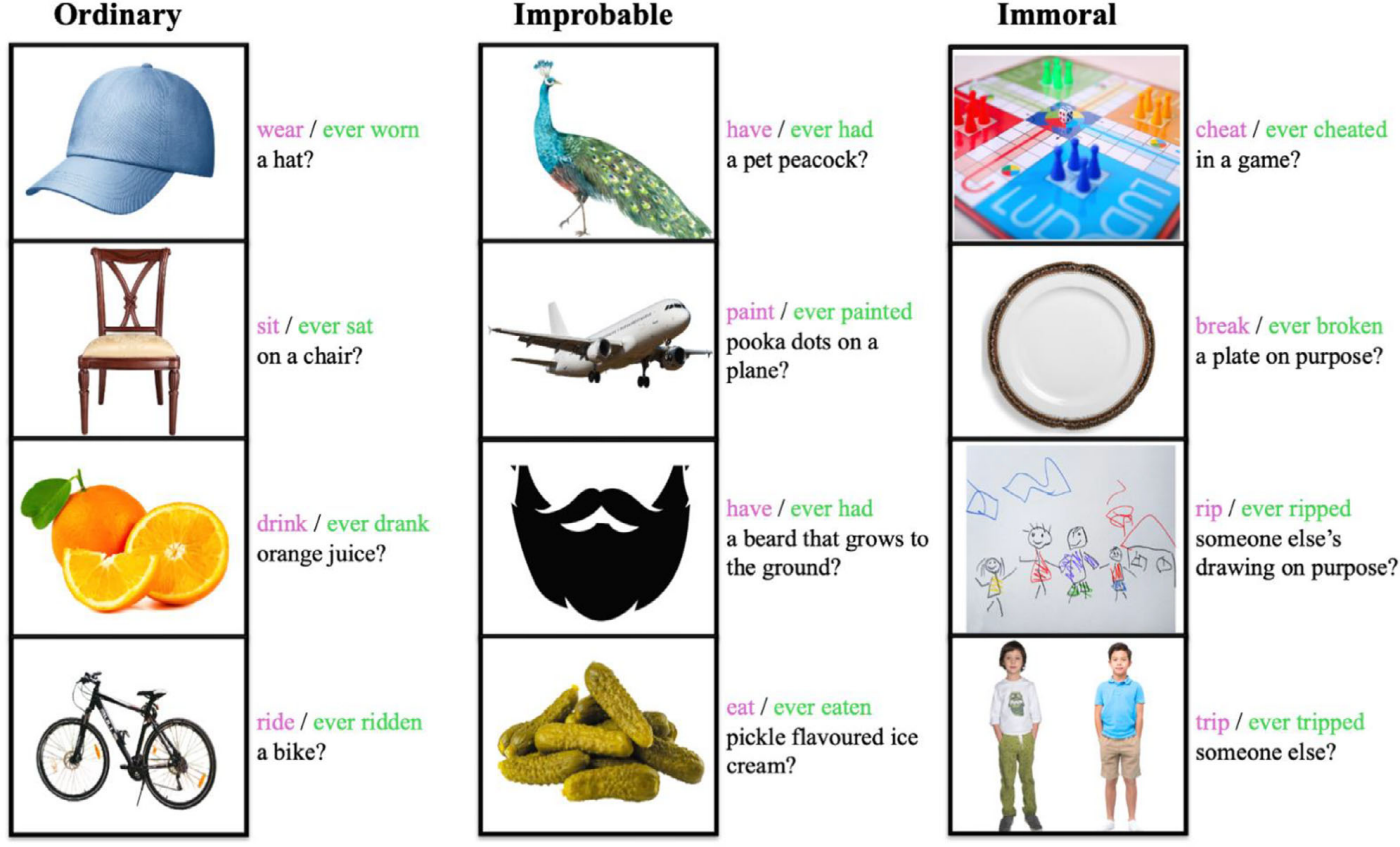
Experiment 1: Stimuli and test questions. *Note*: The pictures and test questions in Experiment 1. Children were asked about ordinary, improbable, and immoral events. In one between‐subjects condition, the questions began with "*In real life, could someone*…,” while in the other condition, the questions began with “*In real life, has someone ever*…”

**Materials and procedure**. Children were randomly assigned to one of two between‐subject conditions; see Figure 1 for the script and stimuli. In one between‐subjects condition, children were first told, “I'm going to tell you a bunch of things, and I want to know if they *could happen* in real life.” Then across a series of 12 test trials, children were asked whether different events could happen in real life (e.g., “In real life, *could someone* have a pet peacock?”); each event had an accompanying picture shown on a laptop screen. In the other between‐subjects condition, children were instead first told, “I'm going to tell you a bunch of things, and I want to know if they *have ever happened* in real life.” They were asked about the same 12 events but judged if they have ever happened (e.g., “In real life, *has someone ever* had a pet peacock?”).

The 12 trials consisted of ordinary, improbable, and immoral events, with four events of each type. The ordinary events were wearing a hat, sitting on a chair, drinking orange juice, and riding a bike. The improbable events were having a pet peacock, painting polka dots on a plane, having a beard that grows to the ground, and eating pickle‐flavored ice cream. We took these from earlier papers, finding that children deny the possibility of improbable events (Shtulman and Carey 2007; Nancekivell and Friedman 2017). The immoral events were cheating in a game, breaking a plate on purpose, ripping someone else's drawing on purpose, and tripping someone. Some of these events were simplified from Shtulman and Phillips (2018), while others were chosen because previous work suggests that children recognize their immorality (e.g., Marlow et al. 2023; Vaish et al. 2011). Children either completed the trials in the following order, or in the reverse order: peacock, hat, cheat, chair, break, plane, beard, rip, orange, trip, pickle, bicycle. The improbable events were ones that children claimed to be impossible in previous papers (Shtulman and Carey 2007; Nancekivell and Friedman 2017)

### 3.2 Results

**FIGURE 2:**
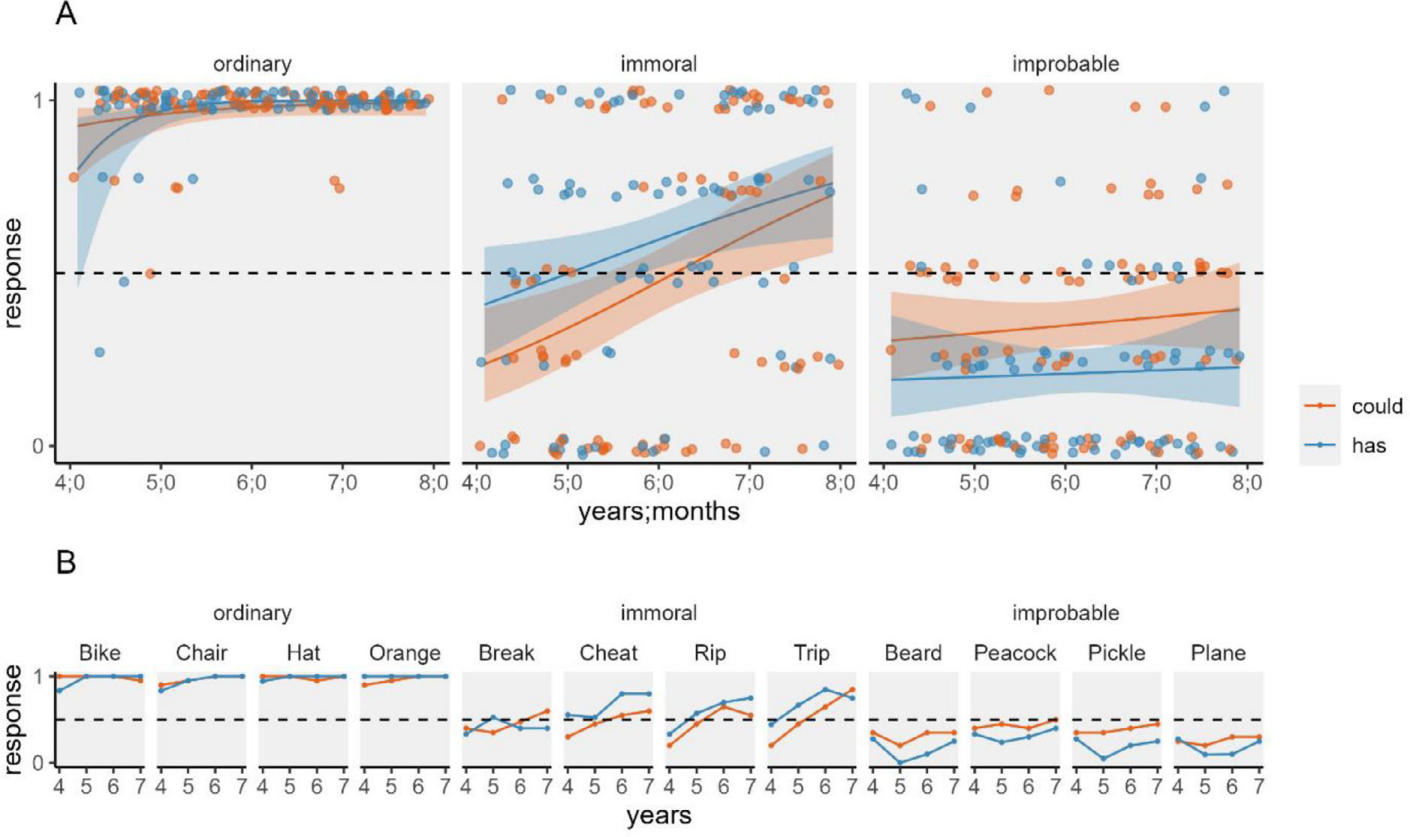
Experiment 1: Judgments that events could happen or had happened. *Note*: Children answered yes (1)/no (0) questions about whether events could happen or had ever happened. The top panel (A) shows plots generated from the GEE model with predicted values derived using *ggeffects*; lines show predicted mean scores, bands show 95% confidence intervals, and jittered points show individual participants’ mean responses. The lower panel (B) shows children's mean responses for the particular items of each type at each age in years.

Figure 2 shows children's responses (“yes” coded 1; “no” coded 0), including both the overall pattern of responses and children's mean responses for individual items. We entered responses into a GEE model for binary data, with condition (could and has), item type (ordinary, immoral, and improbable), and age in months as predictors. It revealed a main effect of item type, *F*(2, 1894) = 56.68, *p* < 0.001, a main effect of age, *F*(1, 1894) = 14.67, *p* < 0.001, and no main effect of condition, *F*(1, 1894) = 1.58, *p* = 0.208. There was a significant interaction between item type and condition, *F*(2, 1894) = 7.61, *p* < 0.001, a significant interaction between item type and age, *F*(2, 1894) = 6.97, *p* < 0.001, and no significant interaction between condition and age, *F*(1, 1894) = 1.59, *p* = 0.218. There was no significant interaction between item type, condition, and age, *F*(2, 1894) = 2.97, *p* = 0.226.

The main effect of item type resulted because children were more likely to see ordinary items as possible compared to both immoral, odds ratio (OR) = 103.85, *t*(1894) = 7.60, *p* < 0.001 (*p* values holm‐adjusted for three tests), and improbable items, OR = 322.48, *t*(1894) = 9.28, *p* < 0.001, and more likely to see immoral items as possible compared to improbable ones, OR = 3.11, *t*(1894) = 6.98, *p* < 0.001. The interaction between item type and condition resulted because children were more likely to say improbable events could happen than to say they had ever happened, OR = 2.03, *t*(1894) = 2.88, *p* = 0.004, whereas condition did not significantly affect responses for ordinary, OR = 0.17, *t*(1894) = 1.49, *p* = 0.136, and immoral events, OR = 0.61, *t*(1894) = 1.92, *p* = 0.055 (*p* values adjusted for three tests). Also, although condition did not significantly affect judgments for immoral events, the direction of the difference between the two judgments fell in opposite directions, which likely contributed to the interaction.

The interaction between item type and age resulted because older children were more likely than younger children to affirm possibility for ordinary events, *t*(1894) = 3.10, *p* = 0.004, *b* = 0.12 (*p* values holm‐adjusted for three tests), and immoral events, *t*(1894) = 4.01, *p* < 0.001, *b* = 0.04, whereas there was no effect of age for improbable events, *t*(1894) = 0.64, *p* = 0.526, *b* = 0.01.

Finally, to examine whether children mostly affirmed or mostly denied the possibility for items of each type, we examined whether 95% confidence intervals (CI) overlapped with chance. Given the findings above, for ordinary and immoral items, we examined the ages at which 95% CI departed from chance but without distinguishing between the two kinds of judgments. For improbable events, by contrast, we did not look at age but did distinguish between judgments of possibility and actuality.

With ordinary items, even the youngest four (i.e., Age 4;0) mostly affirmed possibility (95% CI [0.68, 0.95]). With immoral events, children up to Age 4;11 mostly denied the possibility (95% CI [0.33, 0.49]) and those from Age 6;4 (95% CI [0.51, 0.64]) mostly affirmed it. With improbable events, children mostly denied possibility when judging both what could happen (95% CI [0.29, 0.42]) and when it had happened (95% CI [0.15, 0.28]).

### 3.3 Discussion

Children responded similarly regardless of whether they were asked if events could happen or if events had ever happened. With both judgments, they mostly judged that ordinary events were possible, mostly judged that improbable events were impossible, and with age shifted from denying to affirming the possibility of immoral events. We also found that with age, children increasingly admitted the possibility of ordinary and immoral events, regardless of judgment type, whereas there were no developmental changes in children's responses for improbable events. Only one difference between judgment types emerged: Children were more likely to say that improbable events could happen than to say they had ever happened.

These findings suggest that children's denials are not just limited to “could questions” and also arise in their judgments of actuality. To further investigate this pattern, we probed judgments of actuality in our next experiment in another way. We told children about assertions that an event had happened and asked whether the assertions could be true. This next experiment focused on children aged 5–7 and did not include those aged 4; their responses to ordinary items suggested that they were more susceptible than older children to task demands.

## 4 Experiment 2

### 4.1 Methods

**Participants**. We tested sixty‐one 5–7‐year‐olds (*M*_age_ = 6;6, range = 5;0–7;11, 34 female and 27 male). An additional four children were seen but excluded from analysis: two because of experimental error, and two because they responded to fewer than half of the test questions.

**Materials and procedure**. We used the same procedure as Experiment 1 but with a new testing script. For each trial, participants were told someone claimed to have done something and were then asked if the claim could be true. For instance, “*Somebody said they had a pet peacock. Could that be true?*”

### 4.2 Results

**FIGURE 3:**
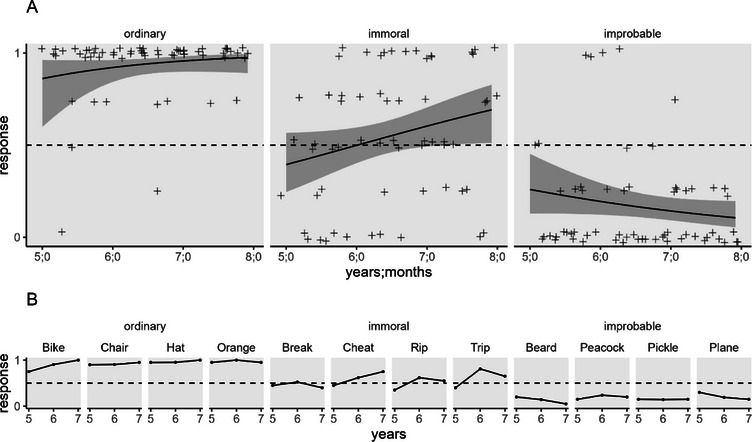
Experiment 2: Judgments that the informant's testimony could be true. *Note*: Children answered yes (1)/no (0) questions about whether claims about ordinary, immoral, and improbable events could be true. The top panel (A) shows plots generated from the GEE model with predicted values derived using *ggeffects*; lines show predicted mean scores, bands show 95% confidence intervals, and jittered points show individual participants’ mean responses. The lower panel (B) shows children's mean responses for the particular items of each type at each age in years.

Figure 3 shows children's responses (“yes” coded 1; “no” coded 0), including both the overall pattern of responses and children's mean responses for individual items. We entered children's responses into a GEE model for binary data with item type (ordinary, immoral, and improbable) and age in months as predictors. It revealed a main effect of item type, *F*(2, 726) = 57.88, *p* < 0.001, no main effect of age, *F*(1, 726) = 1.28, *p* = 0.259, and a significant interaction, *F*(2, 726) = 3.28, *p* = 0.038. Overall, children were more likely to say claims could be true if they were asked about ordinary events than both immoral, OR = 13.12, *t*(726) = 7.12, *p* < 0.001 (*p* values holm‐adjusted for three tests), and improbable ones, OR = 79.89, *t*(726) = 10.75, *p* < 0.001, and more likely to say claims could be true if there were asked about immoral events than improbable ones, OR = 6.09, *t*(726) = 5.98, *p* < 0.001. Although the interaction between type and age was significant, the effect of age on each item type was not significant, *p*s ≥ 0.167.

To examine whether children mostly affirmed or mostly denied possibility for items of each type, we again examined whether the 95% CI overlapped with chance. We did not consider age in these comparisons given that it did not significantly affect responses for any item type. Children mostly affirmed the possibility for ordinary items (95% CI [0.89, .97]), responded at chance for immoral items (95% CI [0.46, 0.64]), and mostly denied the possibility for improbable items (95% CI [0.11, 0.24]).

**Exploratory comparison across experiments**. To further compare children's judgments of actuality and possibility, we also conducted an exploratory analysis comparing children's judgments about which statements could be true from this experiment (actuality) with judgments about which events could happen from the first experiment (possibility). Because this experiment included children aged 5 years and older, we excluded the 4‐year‐olds from the first experiment.

We examined children's responses using a GEE model with judgment (could be true, could happen), item type (ordinary, immoral, and improbable), and age in months as predictors. All three main effects were significant: judgment, *F*(1, 1438) = 8.88, *p* = 0.003; item type, *F*(2, 1438) = 85.30, *p* < 0.001; and age, *F*(1,1438) = 4.42, *p* = 0.036. These effects were qualified by two‐way interactions between item type and judgment, *F*(2, 1438) = 4.03, *p* = 0.018, and between item type and age, *F*(2, 1438) = 3.77, *p* = 0.023. The interaction between judgment and age was not significant, *F*(1, 1438) = 0.48, *p* = 0.490, and the three‐way interaction was also not significant, *F*(2,1438) = 0.71, *p* = 4.90.

The main effect of judgment resulted because children were overall more likely to give positive responses when judging if events could happen (Experiment 1) than when judging if statements could be true (Experiment 2). The main effect of item type resulted because (echoing the earlier analyses) children more often gave positive responses for ordinary than both immoral events, OR = 26.82, *t*(1438) = 8.74*, p* < 0.001 (*p* values holm‐adjusted for three tests), and improbable ones, OR = 100.01, *t*(1438) = 12.42, *p* < 0.001, and for immoral events than improbable ones, OR = 3.73, *t*(1438) = 6.82, *p* < 0.001. The interaction between these factors arose because whereas children gave more positive responses for events than statements with ordinary, OR = 4.21, *t*(1438) = 2.09, *p* = 0.037 (*p* values holm‐adjusted for three tests), and improbable items, OR = 2.72, *t*(1438) = 3.32, *p* = 0.001, responses did not differ for immoral ones, OR = 1.01, *t*(1438) = 0.03, *p* = 0.973.

Finally, the two‐way interaction between item type and age resulted because there was an age‐related increase in positive responses for immoral items, *t*(1438) = 2.59, *p* = 0.029, *b* = 0.04 (*p* values holm‐adjusted for three tests), whereas age did not significantly affect responses for ordinary and improbable items, *p*s ≥ 0.223.

### 4.3 Discussion

Children's responses resembled those from the first experiment, even though they were asked a novel type of test question, about whether claims about different events could be true. Children mostly judged that statements describing ordinary events were true, that statements about improbable events were false, and their judgments about immoral events fell between these extremes. Our exploratory comparison of responses across the two experiments found that, if anything, children were *more* skeptical when asked if statements could be true than when asked if events could happen.

## 5 General Discussion

In two experiments, we compared children's judgments of possibility with their judgments of actuality. In the first experiment, children aged 4–7 were asked about whether ordinary, immoral, and improbable events could happen, or about whether these events had ever happened. In the second experiment, children aged 5–7 were told that people had claimed the events had happened and then judged whether these claims could be true. Regardless of the questions asked, children responded similarly: Whereas they admitted the possibility of ordinary events, they often denied this for immoral events, with even stronger denials for improbable events.

Our findings suggest that children's surprising possibility denials can be taken at face value and are unlikely to arise from quirks in how they understand and interpret questions about possibility. In most studies investigating these judgments, children were asked about whether events could happen in real life. Because these questions can either be taken to refer to true possibility (what can happen) or to permissibility (what should happen), a lingering concern was that children's denials might indicate beliefs that events *should not* happen rather than beliefs that events are impossible. The present findings dispel this concern, as children's possibility judgments largely matched their judgments of actuality. This said, the face value and ambiguous meaning accounts are not exhaustive, and other accounts might explain the findings. For instance, children could have misunderstood the test questions in some other way, though it is unclear what misunderstandings might bring children to reach a common misinterpretation of all three questions.

Before discussing the further implications of our findings, it is worth acknowledging a potential concern. In contrast with most other studies examining children's surprising possibility denials, we did not find age‐related increases in children's judgments that improbable events could happen. One potential explanation is that we tested children across a somewhat narrow range of ages. Other papers have tested children aged 4–8 (Lane et al. 2016; Nissel et al. 2024; Shtulman and Carey 2007) or 5–9 (Goulding et al. 2024), whereas we tested children aged 4–7 in our first experiment and aged 5–7 in our second. But this explanation may not be credible, since we found age‐related increases for ordinary and immoral events in the first experiment, and the increase for immoral events was also significant in the analysis combining data from 5‐ to 7‐year‐olds across the experiments. Another possibility is that the effect of age was an accident of item selection—we only asked children about four items of each type, and so perhaps the improbable items we chose are particularly unexpected for children in our age range. A further contributing factor might be that some improbable items featured less familiar words and items (e.g., polka dots, peacock) than the ordinary and immoral events (though this aspect of our design is likely consistent with previous experiments).

### 5.1 Implications

The similarity in responses across questions suggests that a single procedure, or set of procedures, for assessing possibility contributed to children's judgments irrespective of whether they were asked about what could happen, what has happened, or whether an utterance might be true. It also implies that children's modal judgments are not particularly sensitive to framing effects and surface features of the way questions are posed (e.g., Flusberg et al. 2024; Hsee 1996), and are instead robust to different prompts, similar to adults’ modal judgments (Acierno et al. 2022; Phillips and Cushman 2017). This said, there were some differences between the judgments. In both the first experiment and the exploratory analysis in the second one, children were somewhat more likely to affirm possibility than actuality for improbable items. For instance, they were more likely to say a person could have a pet peacock than to say someone had ever had such a pet. This difference reinforces the conclusion that children's *could* judgments really do reflect their thinking about possibility, since many possible events have never actually happened.

Previous work has outlined different accounts of how children might assess whether an outcome is possible: by simulating circumstances under which it could occur (Shtulman and Carey 2007); by identifying causal mechanisms that could bring it about (Goulding et al. 2022; Shtulman et al. 2024); by assessing how similar it is to known events (Goulding and Friedman 2021); or by basing judgments of what could happen on expectations about what should happen (Shtulman and Phillips 2018; also see Phillips and Cushman 2017). While these accounts are not mutually exclusive, our findings provide some support for the last account. It is the only account to specifically explain why children should deny the possibility of immoral events. The present findings reinforce this surprising finding: Younger children not only denied that immoral events could happen, but also denied that they ever had happened, even though we asked about immoral events that they were likely to have personally encountered.

Additional work will be needed to confirm that children assess the possibility the same way across diverse questions. Further support for this conjecture would be that *individual* children respond similarly to all three questions. Our experiments do not speak to this since our participants were each asked just one type of question. Children who are asked about both possibility and actuality should provide the same answer if perceptions of actuality inform judgments of possibility and vice versa. Indeed, children asked about permissibility should also provide the same answer, if early modal judgments conflate all three dimensions (i.e., events that could not happen have not happened and should not happen).

The overall message from our findings, then, is that children's surprising possibility denials can be taken at face value—when children deny that a person can drink onion juice, they have concluded this event cannot happen. These denials may be less surprising, though, if we contrast judgments made on‐the‐fly with those that reflect established beliefs.

Children's denials for many improbable events, like the possibility of drinking onion juice, may be transient judgments made on‐the‐fly. That is, when children are asked about such situations, they probably do not have many established beliefs about them. On the contrary, children may conclude that drinking onion juice is impossible precisely because they *lack* relevant beliefs and knowledge—perhaps because they are unable to think of similar known events (Goulding and Friedman 2021) or because they cannot envision the circumstances that would allow it to happen (Shtulman and Carey 2007). Viewed this way, children's denials of conceptually improbable events may be less surprising. While it would be surprising if children held the long‐established belief that drinking onion juice is impossible, their denials do not show or imply this.

On‐the‐fly judgments, though, do not make denials for immoral events easier to understand since children have firsthand experience with such events. For this domain of events, things work the opposite way: Children's denials may stem from longstanding beliefs rather than on‐the‐fly intuitions. For instance, the longstanding belief *people cannot steal *may somehow support both the conclusion that stealing is impermissible and that it is impossible. This account suggests that children exhibit a systematic kind of memory failure. If children recalled their experiences with immoral events, they would know these events could happen and indeed have happened.

Why might children fail to consult their memories when asked about possibility and actuality? Perhaps it is because we asked children about people in general (e.g., “has someone ever…”), and this way of asking the question might invite children to consider what is possible, whereas asking children about themselves (e.g., “have you ever”) or about people they know (“has someone you know ever …”) might encourage them to consult their memories. It is important to note that this kind of memory neglect is *not* entirely unique to children, since adults, too, deny the possibility of immoral events when responding under time pressure (Phillips and Cushman 2017). The adult findings suggest that when people consider what is possible, they default to considering permissibility—either before consulting their memories, or perhaps alongside. Our findings suggest that considerations of permissibility affect how we think about actuality as well, though it remains an open question whether this effect persists beyond childhood. When forced to respond quickly, even adults might deny that immoral events have ever happened or that assertions about immoral acts could be true.

## Author Contributions

**Mopreet Pabla**: conceptualization, methodology, investigation, writing – original draft, visualization, formal analysis. **Andrew Shtulman**: conceptualization, writing – review and editing, methodology. **Ori Friedman**: conceptualization, funding acquisition, writing – review and editing, methodology, formal analysis.

## Ethics Statement

This research was approved by the Office of Research Ethics at the University of Waterloo (Project 30395: Social Understanding in Children).

## Conflicts of Interest

The authors declare no conflicts of interest.

## Funding

This research was supported by a grant from the Natural Sciences and Engineering Research Council of Canada awarded to O.F.

## Data Availability

Data and code can be downloaded at the Open Science Foundation at https://osf.io/dx568/.

## Endnotes

1 Young children are also often inflexible in recognizing that norms could differ (e.g., Komatsu and Galotti 1986; Levy et al. 1995; Lockhart et al. 1977; Miller et al. 2000; Nicholls and Thorkildsen 1988). For instance, younger children deny that people could eat ice cream with their hands rather than with a spoon. Our focus here is instead on children's views about the possibility of events not norms.

2 Concerns from the ambiguous meaning account about children's interpretation of “could” do not apply to its use in this question since it would be difficult to construe the question as asking about something besides the potential truth of the assertion.

3 There are some events for which the face value account would not predict correspondences across the questions. One example is questions that refer to possibilities restricted to particular time periods. For instance, most adults would likely say “no” if asked “Could the Queen of France be living in Versailles?” since currently there is no Queen of France. But they might say “yes” if asked “Has the Queen of France ever lived in Versailles?”
